# Angioedema with haloperidol - case report

**DOI:** 10.1192/j.eurpsy.2021.1284

**Published:** 2021-08-13

**Authors:** A. Costa, S. Jesus, J. Alcafache

**Affiliations:** Psiquiatria E Saúde Mental, Centro Hospitalar do Baixo Vouga, Aveiro, Portugal

**Keywords:** Angioedema, Anaphylactic reaction, Haloperidol

## Abstract

**Introduction:**

Haloperidol is a high-potency first generation antipsychotic and one of the most frequently used antipsychotic medications. It is a potent central antagonist of type 2 dopamine receptors, with low alpha 1 adrenergic activity and has no antihistamine or anti-cholinergic activity. It is a widely used drug with proven efficacy. Angioedema is a very rare side effect, occurring in <1% of cases.

**Objectives:**

Case report and reflection on its etiology

**Methods:**

A Pubmed search was performed with the MeSH terms “haloperidol” and “Anaphylactic reactions”. Relevant articles obtained from the respective bibliographic references were also consulted.

**Results:**

The following case describes the development of angioedema in a patient with an acute confusional syndrome on the second haloperidol IM administration for symptomatic control of agitation. Angioedema has been reported as an adverse effect of various antipsychotics such as clozapine, risperidone, ziprazidone and chlorpromazine, however, resulting from haloperidol administration is rare.

**Conclusions:**

In long-term formulations sensitization testing is especially important but a single prior administration is not sufficient, a second controlled administration is essential to avoid this kind of fatal reactions.

